# l-Histidinium *p*-toluene­sulfonate

**DOI:** 10.1107/S1600536813011161

**Published:** 2013-04-30

**Authors:** Srinivasan Muralidharan, Perumal Nagapandiselvi, Thothadri Srinivasan, Rengasamy Gopalakrishnan, Devadasan Velmurugan

**Affiliations:** a Department of Physics, Anna University, Chennai 600 025, India; b Centre of Advanced Study in Crystallography and Biophysics, University of Madras, Guindy Campus, Chennai 600 025, India

## Abstract

In the title salt, C_6_H_10_N_3_O_2_^+^·C_7_H_7_O_3_S^−^, the imidazole ring makes a dihedral angle of 70.93 (12)° with the plane of the toluene ring. In the crystal, the ions are linked *via* N—H⋯O and weak C—H⋯O hydrogen bonds forming two-dimensional networks lying parallel to (001). These networks are linked *via* C—H⋯π inter­actions, forming a three-dimensional structure.

## Related literature

For related structures of 4-toluene­sulfonate salts, see: Koshima *et al.* (2004 ▶); Biradha & Mahata (2005 ▶); Sivakumar *et al.* (2012 ▶). For the structure of l-histidine, see: Madden *et al.* (1972 ▶); Andra *et al.* (2010 ▶).
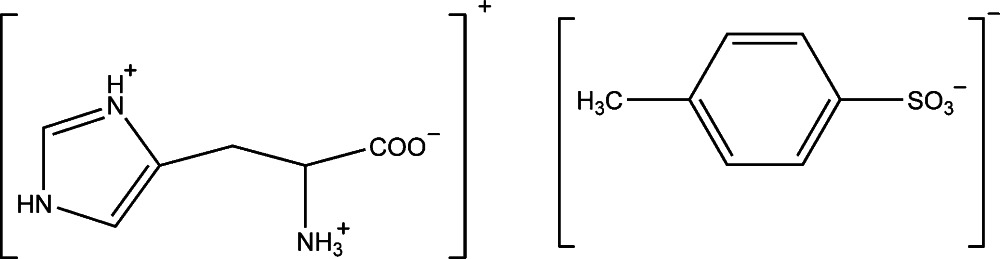


## Experimental

### 

#### Crystal data

C_6_H_10_N_3_O_2_^+^·C_7_H_7_O_3_S^−^
*M* *_r_* = 327.36
Orthorhombic, 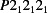

*a* = 5.2700 (2) Å
*b* = 7.3691 (3) Å
*c* = 38.2042 (14) Å
*V* = 1483.67 (10) Å^3^
*Z* = 4
Mo *K*α radiation
μ = 0.25 mm^−1^
*T* = 293 K
0.30 × 0.25 × 0.20 mm

#### Data collection

Bruker SMART APEXII area-detector diffractometer
Absorption correction: multi-scan (*SADABS*; Bruker, 2008 ▶) *T*_min_ = 0.930, *T*_max_ = 0.952
8400 measured reflections
3638 independent reflections
3533 reflections with *I* > 2σ(*I*)
*R*_int_ = 0.022

#### Refinement

*R*[*F* ^2^ > 2σ(*F* ^2^)] = 0.038
*wR*(*F* ^2^) = 0.094
*S* = 1.20
3638 reflections
212 parameters
H atoms treated by a mixture of independent and constrained refinement
Δρ_max_ = 0.22 e Å^−3^
Δρ_min_ = −0.29 e Å^−3^
Absolute structure: Flack (1983 ▶), 1479 Friedel pairs
Flack parameter: 0.07 (7)

### 

Data collection: *APEX2* (Bruker, 2008 ▶); cell refinement: *SAINT* (Bruker, 2008 ▶); data reduction: *SAINT*; program(s) used to solve structure: *SHELXS97* (Sheldrick, 2008 ▶); program(s) used to refine structure: *SHELXL97* (Sheldrick, 2008 ▶); molecular graphics: *ORTEP-3 for Windows* (Farrugia, 2012 ▶); software used to prepare material for publication: *SHELXL97* and *PLATON* (Spek, 2009 ▶).

## Supplementary Material

Crystal structure: contains datablock(s) global, I. DOI: 10.1107/S1600536813011161/su2589sup1.cif

Structure factors: contains datablock(s) I. DOI: 10.1107/S1600536813011161/su2589Isup2.hkl

Supplementary material file. DOI: 10.1107/S1600536813011161/su2589Isup3.cml

Additional supplementary materials:  crystallographic information; 3D view; checkCIF report

## Figures and Tables

**Table 1:** Hydrogen-bond geometry (Å, °) *Cg*1 and *Cg*2 are the centroids the C1–C6 and N1/N2/C8–C10 rings, respectively.

*D*—H⋯*A*	*D*—H	H⋯*A*	*D*⋯*A*	*D*—H⋯*A*
N1—H1⋯O2^i^	0.86	2.00	2.828 (2)	160
N2—H2*A*⋯O3^ii^	0.86	1.89	2.746 (2)	175
N3—H3*B*⋯O4^iii^	0.96 (3)	1.80 (3)	2.755 (2)	176 (2)
N3—H3*C*⋯O4^iv^	0.83 (3)	2.10 (3)	2.896 (2)	161 (2)
N3—H3*D*⋯O3^i^	0.92 (2)	2.15 (2)	3.000 (2)	153.4 (19)
C8—H8⋯O1^v^	0.93	2.41	2.967 (3)	118
C9—H9⋯O5^vi^	0.93	2.47	3.062 (3)	122
C6—H6⋯*Cg*2	0.93	2.73	3.515 (2)	143
C8—H8⋯*Cg*1^ii^	0.93	2.71	3.394 (2)	131

Symmetry codes: (i) 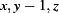; (ii) 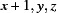; (iii) 
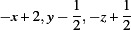
; (iv) 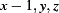; (v) 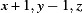; (vi) 
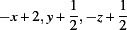
.

**Fig. 1.**
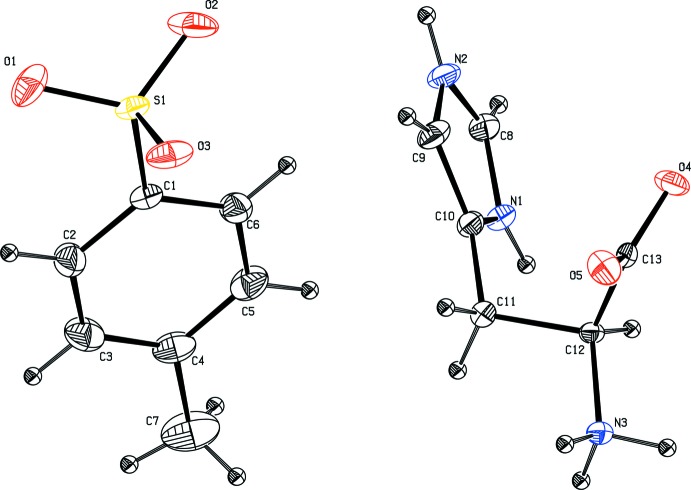
The molecular structure of the title salt, with atom labelling. Displacement ellipsoids are drawn at the 30% probability level.

**Fig. 2.**
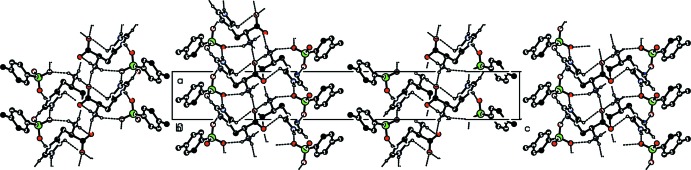
The crystal packing of the title compound viewed along the b axis. The hydrogen bonds are shown as dashed lines (see Table 1 for details; H-atoms not involved in hydrogen bonds have been omitted for clarity).

## supplementary crystallographic information

### Comment

The asymmetric unit of the title compound, Fig. 1, contains an L-histidinium cation and a 4-toluenesulfonate anion. The histidine molecule exists as an histidinium ion due to the protonation at the N atom of the imidazole ring. The 4-toluenesulfonic acid exists as a 4-toluenesulfonate since the proton is transferred to the amino acid.

In the crystal, the imidazole ring (N1/N2/C8-C10) makes a dihedral angle of 70.93 (12)° with the toluene ring (C1-C6).

In the crystal, the ions are linked via N-H···O and weak C-H···O hydrogen bonds forming two-dimensional networks lying parallel to (001); see Table 1 and Fig. 2. These networks are linked via C-H···π interactions forming a three-dimensional structure.

### Experimental

L-histidine and 4-toluenesulfonic acid were mixed in an equimolar (1:1) ratio using distilled water as solvent and stirred for 1 h, giving a clear solution. The solution was filtered into a clean beaker and optimally closed and kept at room temperature for slow evaporation. After a period of 10 days, block-like colourless crystals suitable for X-ray diffraction analysis were obtained.

### Refinement

The NH_3_ H atoms were located in a difference Fourier map and refined freely. The NH H atoms and the C-bound H atoms were positioned geometrically and refined using a riding model: N—H = 0.86 Å, C—H = 0.93 and 0.98 Å for CH(aromatic) and CH(methine) H atoms, respectively, and 0.96 Å for CH_2_ and CH_3_ H atoms, with U_iso_(H) = 1.5U_eq_(C-methyl) and = 1.2U_eq_(N,C) for other H atoms.

### Crystal data

**Table:**

C_6_H_10_N_3_O_2_^+^·C_7_H_7_O_3_S^−^	*F*(000) = 688
*M**_r_* = 327.36	*D*_x_ = 1.466 Mg m^−^^3^
Orthorhombic, *P*2_1_2_1_2_1_	Mo *K*α radiation, λ = 0.71073 Å
Hall symbol: P 2ac 2ab	Cell parameters from 3638 reflections
*a* = 5.2700 (2) Å	θ = 2.1–28.3°
*b* = 7.3691 (3) Å	µ = 0.25 mm^−^^1^
*c* = 38.2042 (14) Å	*T* = 293 K
*V* = 1483.67 (10) Å^3^	Block, colourless
*Z* = 4	0.30 × 0.25 × 0.20 mm

### Data collection

**Table:**

Bruker SMART APEXII area-detector diffractometer	3638 independent reflections
Radiation source: fine-focus sealed tube	3533 reflections with *I* > 2σ(*I*)
Graphite monochromator	*R*_int_ = 0.022
ω and φ scans	θ_max_ = 28.3°, θ_min_ = 2.1°
Absorption correction: multi-scan (*SADABS*; Bruker, 2008)	*h* = −6→6
*T*_min_ = 0.930, *T*_max_ = 0.952	*k* = −9→6
8400 measured reflections	*l* = −41→50

### Refinement

**Table:**

Refinement on *F*^2^	Secondary atom site location: difference Fourier map
Least-squares matrix: full	Hydrogen site location: inferred from neighbouring sites
*R*[*F*^2^ > 2σ(*F*^2^)] = 0.038	H atoms treated by a mixture of independent and constrained refinement
*wR*(*F*^2^) = 0.094	*w* = 1/[σ^2^(*F*_o_^2^) + (0.0378*P*)^2^ + 0.5138*P*] where *P* = (*F*_o_^2^ + 2*F*_c_^2^)/3
*S* = 1.20	(Δ/σ)_max_ = 0.014
3638 reflections	Δρ_max_ = 0.22 e Å^−^^3^
212 parameters	Δρ_min_ = −0.29 e Å^−^^3^
0 restraints	Absolute structure: Flack (1983), 1479 Friedel pairs
Primary atom site location: structure-invariant direct methods	Flack parameter: 0.07 (7)

### Special details

**Table:**

Geometry. All esds (except the esd in the dihedral angle between two l.s. planes) are estimated using the full covariance matrix. The cell esds are taken into account individually in the estimation of esds in distances, angles and torsion angles; correlations between esds in cell parameters are only used when they are defined by crystal symmetry. An approximate (isotropic) treatment of cell esds is used for estimating esds involving l.s. planes.
Refinement. Refinement of F^2^ against ALL reflections. The weighted R-factor wR and goodness of fit S are based on F^2^, conventional R-factors R are based on F, with F set to zero for negative F^2^. The threshold expression of F^2^ > 2sigma(F^2^) is used only for calculating R-factors(gt) etc. and is not relevant to the choice of reflections for refinement. R-factors based on F^2^ are statistically about twice as large as those based on F, and R- factors based on ALL data will be even larger.

### Fractional atomic coordinates and isotropic or equivalent isotropic displacement parameters (Å^2^)

**Table:**

	*x*	*y*	*z*	*U*_iso_*/*U*_eq_	
C1	0.5686 (4)	0.8856 (3)	0.08354 (5)	0.0267 (4)	
C2	0.3546 (5)	0.8904 (3)	0.06249 (6)	0.0378 (5)	
H2	0.2447	0.9891	0.0633	0.045*	
C3	0.3067 (5)	0.7455 (4)	0.04012 (6)	0.0445 (6)	
H3	0.1645	0.7493	0.0257	0.053*	
C4	0.4653 (5)	0.5955 (3)	0.03871 (6)	0.0410 (5)	
C5	0.6786 (5)	0.5940 (3)	0.05970 (6)	0.0413 (5)	
H5	0.7888	0.4956	0.0588	0.050*	
C6	0.7307 (4)	0.7383 (3)	0.08214 (6)	0.0352 (5)	
H6	0.8747	0.7355	0.0962	0.042*	
C7	0.4046 (8)	0.4355 (4)	0.01540 (8)	0.0698 (9)	
H7A	0.2604	0.4640	0.0010	0.105*	
H7B	0.3667	0.3315	0.0296	0.105*	
H7C	0.5479	0.4093	0.0007	0.105*	
C8	1.2390 (4)	0.5424 (3)	0.13142 (5)	0.0305 (4)	
H8	1.3637	0.5258	0.1145	0.037*	
C9	1.0008 (4)	0.6656 (3)	0.17226 (5)	0.0312 (4)	
H9	0.9349	0.7496	0.1880	0.037*	
C10	0.9162 (4)	0.4935 (2)	0.16686 (5)	0.0240 (4)	
C11	0.7122 (4)	0.3892 (2)	0.18479 (5)	0.0242 (4)	
H11A	0.6039	0.3330	0.1673	0.029*	
H11B	0.6089	0.4719	0.1985	0.029*	
C12	0.8209 (3)	0.2411 (2)	0.20905 (4)	0.0190 (3)	
H12	0.9473	0.1693	0.1963	0.023*	
C13	0.9463 (3)	0.3308 (2)	0.24112 (5)	0.0217 (4)	
N1	1.0671 (3)	0.4207 (2)	0.14111 (4)	0.0261 (3)	
H1	1.0525	0.3132	0.1326	0.031*	
N2	1.2021 (4)	0.6914 (2)	0.15002 (5)	0.0325 (4)	
H2A	1.2904	0.7892	0.1484	0.039*	
N3	0.6079 (3)	0.1216 (2)	0.22022 (4)	0.0227 (3)	
O1	0.4968 (4)	1.2199 (2)	0.10150 (5)	0.0513 (5)	
O2	0.8939 (3)	1.0777 (2)	0.11806 (5)	0.0564 (5)	
O3	0.5067 (4)	0.9933 (2)	0.14701 (4)	0.0448 (4)	
O4	1.1834 (3)	0.3507 (2)	0.23929 (4)	0.0344 (4)	
O5	0.8051 (3)	0.3822 (2)	0.26480 (4)	0.0340 (3)	
S1	0.62131 (10)	1.05973 (6)	0.114496 (13)	0.02896 (12)	
H3D	0.534 (4)	0.064 (3)	0.2015 (6)	0.027 (5)*	
H3B	0.673 (5)	0.024 (4)	0.2341 (7)	0.037 (7)*	
H3C	0.487 (5)	0.173 (4)	0.2300 (6)	0.036 (7)*	

### Atomic displacement parameters (Å^2^)

**Table:**

	*U*^11^	*U*^22^	*U*^33^	*U*^12^	*U*^13^	*U*^23^
C1	0.0301 (10)	0.0217 (8)	0.0283 (8)	−0.0061 (8)	0.0041 (8)	−0.0026 (7)
C2	0.0372 (12)	0.0342 (10)	0.0421 (11)	0.0033 (9)	−0.0061 (10)	−0.0038 (9)
C3	0.0450 (14)	0.0514 (14)	0.0372 (11)	−0.0084 (12)	−0.0092 (10)	−0.0068 (10)
C4	0.0515 (14)	0.0352 (12)	0.0363 (11)	−0.0120 (10)	0.0078 (10)	−0.0102 (9)
C5	0.0457 (13)	0.0272 (10)	0.0509 (13)	0.0019 (9)	0.0099 (11)	−0.0077 (9)
C6	0.0312 (11)	0.0306 (10)	0.0437 (11)	−0.0002 (9)	−0.0012 (9)	−0.0043 (9)
C7	0.095 (3)	0.0578 (17)	0.0570 (16)	−0.0227 (19)	0.0094 (17)	−0.0293 (14)
C8	0.0280 (10)	0.0273 (9)	0.0361 (10)	−0.0045 (9)	0.0013 (8)	0.0055 (8)
C9	0.0449 (12)	0.0186 (8)	0.0302 (10)	−0.0051 (8)	−0.0014 (9)	−0.0016 (7)
C10	0.0275 (9)	0.0202 (8)	0.0244 (8)	−0.0024 (7)	−0.0016 (8)	0.0021 (6)
C11	0.0245 (9)	0.0210 (8)	0.0273 (8)	−0.0011 (7)	−0.0025 (7)	0.0032 (7)
C12	0.0161 (8)	0.0176 (7)	0.0234 (8)	−0.0030 (6)	0.0006 (6)	0.0000 (6)
C13	0.0214 (9)	0.0139 (7)	0.0300 (9)	0.0024 (6)	−0.0055 (7)	0.0004 (6)
N1	0.0307 (9)	0.0176 (7)	0.0301 (7)	−0.0037 (6)	0.0013 (6)	0.0002 (6)
N2	0.0392 (10)	0.0221 (8)	0.0362 (9)	−0.0119 (7)	−0.0043 (8)	0.0049 (7)
N3	0.0188 (7)	0.0202 (7)	0.0290 (8)	−0.0031 (7)	−0.0001 (7)	0.0008 (6)
O1	0.0761 (14)	0.0229 (7)	0.0550 (10)	0.0084 (8)	−0.0015 (10)	−0.0024 (7)
O2	0.0336 (9)	0.0439 (9)	0.0917 (14)	−0.0153 (8)	0.0063 (9)	−0.0324 (10)
O3	0.0614 (11)	0.0379 (8)	0.0350 (8)	−0.0244 (8)	0.0022 (8)	−0.0085 (6)
O4	0.0192 (7)	0.0312 (7)	0.0529 (9)	−0.0031 (6)	−0.0052 (6)	−0.0140 (6)
O5	0.0322 (8)	0.0386 (8)	0.0312 (7)	0.0045 (6)	−0.0018 (6)	−0.0095 (6)
S1	0.0314 (3)	0.0191 (2)	0.0364 (2)	−0.00777 (18)	0.0038 (2)	−0.00459 (18)

### Geometric parameters (Å, º)

**Table:**

C1—C6	1.382 (3)	C9—H9	0.9300
C1—C2	1.386 (3)	C10—N1	1.374 (2)
C1—S1	1.7671 (19)	C10—C11	1.488 (3)
C2—C3	1.391 (3)	C11—C12	1.542 (2)
C2—H2	0.9300	C11—H11A	0.9700
C3—C4	1.387 (4)	C11—H11B	0.9700
C3—H3	0.9300	C12—N3	1.490 (2)
C4—C5	1.381 (4)	C12—C13	1.541 (2)
C4—C7	1.512 (3)	C12—H12	0.9800
C5—C6	1.393 (3)	C13—O5	1.231 (2)
C5—H5	0.9300	C13—O4	1.260 (2)
C6—H6	0.9300	N1—H1	0.8600
C7—H7A	0.9600	N2—H2A	0.8600
C7—H7B	0.9600	N3—H3D	0.92 (2)
C7—H7C	0.9600	N3—H3B	0.96 (3)
C8—N2	1.322 (3)	N3—H3C	0.83 (3)
C8—N1	1.327 (2)	O1—S1	1.4391 (17)
C8—H8	0.9300	O2—S1	1.4490 (18)
C9—C10	1.360 (3)	O3—S1	1.4652 (17)
C9—N2	1.372 (3)		
			
C6—C1—C2	120.04 (18)	C10—C11—C12	111.95 (15)
C6—C1—S1	119.94 (16)	C10—C11—H11A	109.2
C2—C1—S1	119.85 (16)	C12—C11—H11A	109.2
C1—C2—C3	119.0 (2)	C10—C11—H11B	109.2
C1—C2—H2	120.5	C12—C11—H11B	109.2
C3—C2—H2	120.5	H11A—C11—H11B	107.9
C4—C3—C2	121.8 (2)	N3—C12—C13	110.43 (14)
C4—C3—H3	119.1	N3—C12—C11	108.10 (14)
C2—C3—H3	119.1	C13—C12—C11	109.47 (14)
C5—C4—C3	118.3 (2)	N3—C12—H12	109.6
C5—C4—C7	120.5 (2)	C13—C12—H12	109.6
C3—C4—C7	121.1 (3)	C11—C12—H12	109.6
C4—C5—C6	120.8 (2)	O5—C13—O4	127.17 (18)
C4—C5—H5	119.6	O5—C13—C12	117.22 (16)
C6—C5—H5	119.6	O4—C13—C12	115.52 (17)
C1—C6—C5	120.1 (2)	C8—N1—C10	109.33 (16)
C1—C6—H6	119.9	C8—N1—H1	125.3
C5—C6—H6	119.9	C10—N1—H1	125.3
C4—C7—H7A	109.5	C8—N2—C9	109.36 (17)
C4—C7—H7B	109.5	C8—N2—H2A	125.3
H7A—C7—H7B	109.5	C9—N2—H2A	125.3
C4—C7—H7C	109.5	C12—N3—H3D	111.8 (14)
H7A—C7—H7C	109.5	C12—N3—H3B	109.6 (15)
H7B—C7—H7C	109.5	H3D—N3—H3B	104 (2)
N2—C8—N1	108.10 (18)	C12—N3—H3C	115.8 (18)
N2—C8—H8	125.9	H3D—N3—H3C	104 (2)
N1—C8—H8	125.9	H3B—N3—H3C	112 (2)
C10—C9—N2	106.77 (19)	O1—S1—O2	114.19 (12)
C10—C9—H9	126.6	O1—S1—O3	112.24 (12)
N2—C9—H9	126.6	O2—S1—O3	111.07 (12)
C9—C10—N1	106.42 (17)	O1—S1—C1	107.04 (10)
C9—C10—C11	130.43 (19)	O2—S1—C1	106.56 (10)
N1—C10—C11	123.10 (16)	O3—S1—C1	105.06 (9)
			
C6—C1—C2—C3	0.0 (3)	N3—C12—C13—O5	−39.4 (2)
S1—C1—C2—C3	−175.30 (18)	C11—C12—C13—O5	79.5 (2)
C1—C2—C3—C4	0.9 (4)	N3—C12—C13—O4	143.72 (16)
C2—C3—C4—C5	−1.4 (4)	C11—C12—C13—O4	−97.38 (19)
C2—C3—C4—C7	177.3 (2)	N2—C8—N1—C10	−0.4 (2)
C3—C4—C5—C6	1.0 (4)	C9—C10—N1—C8	0.7 (2)
C7—C4—C5—C6	−177.7 (2)	C11—C10—N1—C8	−176.84 (17)
C2—C1—C6—C5	−0.3 (3)	N1—C8—N2—C9	−0.1 (2)
S1—C1—C6—C5	174.95 (17)	C10—C9—N2—C8	0.6 (2)
C4—C5—C6—C1	−0.2 (3)	C6—C1—S1—O1	157.65 (17)
N2—C9—C10—N1	−0.8 (2)	C2—C1—S1—O1	−27.0 (2)
N2—C9—C10—C11	176.54 (19)	C6—C1—S1—O2	35.1 (2)
C9—C10—C11—C12	−106.9 (2)	C2—C1—S1—O2	−149.61 (18)
N1—C10—C11—C12	70.1 (2)	C6—C1—S1—O3	−82.85 (19)
C10—C11—C12—N3	−169.02 (15)	C2—C1—S1—O3	92.46 (19)
C10—C11—C12—C13	70.64 (19)		

### Hydrogen-bond geometry (Å, º)

Cg1 and Cg2 are the centroids the C1–C6 and N1/N2/C8–C10 rings, respectively.

**Table:**

*D*—H···*A*	*D*—H	H···*A*	*D*···*A*	*D*—H···*A*
N1—H1···O2^i^	0.86	2.00	2.828 (2)	160
N2—H2*A*···O3^ii^	0.86	1.89	2.746 (2)	175
N3—H3*B*···O4^iii^	0.96 (3)	1.80 (3)	2.755 (2)	176 (2)
N3—H3*C*···O4^iv^	0.83 (3)	2.10 (3)	2.896 (2)	161 (2)
N3—H3*D*···O3^i^	0.92 (2)	2.15 (2)	3.000 (2)	153.4 (19)
C8—H8···O1^v^	0.93	2.41	2.967 (3)	118
C9—H9···O5^vi^	0.93	2.47	3.062 (3)	122
C6—H6···*Cg*2	0.93	2.73	3.515 (2)	143
C8—H8···*Cg*1^ii^	0.93	2.71	3.394 (2)	131

Symmetry codes: (i) *x*, *y*−1, *z*; (ii) *x*+1, *y*, *z*; (iii) −*x*+2, *y*−1/2, −*z*+1/2; (iv) *x*−1, *y*, *z*; (v) *x*+1, *y*−1, *z*; (vi) −*x*+2, *y*+1/2, −*z*+1/2.
